# Assessment of PPMV-1 Genotype VI Virulence in Pigeons and Chickens and Protective Effectiveness of Paramyxovirus Vaccines in Pigeons

**DOI:** 10.3390/v16101585

**Published:** 2024-10-09

**Authors:** Esraa E. Hamouda, Amal A. M. Eid, Hagar F. Gouda, Amina A. Dessouki, Ayman H. El-Deeb, Rebecca Daines, Munir Iqbal, Reham M. ElBakrey

**Affiliations:** 1 Avian and Rabbit Medicine Department, Faculty of Veterinary Medicine, Zagazig University, Zagazig 44511, Egypt; esraa.ezat58@yahoo.com (E.E.H.); rehamemara3@gmail.com (R.M.E.); 2 Animal Wealth Development Department (Biostatistics Subdivision), Faculty of Veterinary Medicine, Zagazig University, Zagazig 44511, Egypt; stathagarfathi@gmail.com; 3 Pathology Department, Faculty of Veterinary Medicine, Suez Canal University, Suez Canal, Ismailia 41622, Egypt; aminadessouki@hotmail.com; 4 Virology Department, Faculty of Veterinary Medicine, Cairo University, Giza 11361, Egypt; ayman.vv@gmail.com; 5 Microbiology and Parasitology Department, Faculty of Veterinary Medicine, King Salman International University, Ras Sudr 46612, Egypt; 6 Avian Influenza and Newcastle Disease Group, The Pirbright Institute, Ash Road, Pirbright, Woking GU24 0NF, UK; rebecca.daines@pirbright.ac.uk

**Keywords:** pigeon paramyxovirus serotype 1, pathogenicity, histopathology, immunohistochemistry, vaccine efficacy

## Abstract

Pigeon paramyxovirus serotype 1 (PPMV-1), an antigenic and host variant of avian paramyxovirus Newcastle disease virus (NDV), primarily originating from racing pigeons, has become a global panzootic. Egypt uses both inactivated PPMV-1 and conventional NDV vaccines to protect pigeons from disease and mortality. However, the impact of prevalent strains and the effectiveness of available vaccines in pigeons in Egypt are unclear. This study investigates the virulence of PPMV-1 (Pigeon/Egypt/Sharkia-19/2015/KX580988) and evaluates available paramyxovirus vaccines in protecting pigeons against a PPMV-1 challenge. Ten-day-old specific-pathogen-free (SPF) embryonated chicken eggs infected with this strain exhibited a mean death time (MDT) of 86.4 ± 5.88 h. The intracerebral pathogenicity index (ICPI) in day-old chickens was 0.8, while pigeons experienced an ICPI of 0.96 and an intravenous pathogenicity index (IVPI) of 2.11. These findings classify the strain as virulent and velogenic. Experimental infection of pigeons with this PPMV-1 strain at 10^6^ EID_50_/0.1 mL resulted in a 62.5% mortality rate, displaying nervous and enteric distress. The virus caused extensive lesions in visceral organs, with strong immunohistochemistry signals in all examined organs, indicating the systemic spread of the virus concurrent to its neurotropic and viscerotropic tropism. Furthermore, vaccination using an inactivated PPMV-1 and live NDV LaSota vaccine regimen protected 100% of pigeons against mortality, while with a single NDV LaSota vaccine, it was 62.5%. The PPMV alone or combined with NDV LaSota induced protective levels of haemagglutination inhibition (HI) antibody titres and reduced virus shedding from buccal and cloacal cavities. Based on generalised linear gamma model analysis, both PPMV-1 and NDV LaSota are antigenically comparable by HI. These findings suggest that using both inactivated PPMV-1 (G-VI) and live attenuated NDV (LaSota) vaccines is an effective prophylactic regimen for preventing and controlling PPMV-1 and NDV in pigeons, thereby reducing the risk of interspecies transmission.

## 1. Introduction

Pigeons (*Columba livia*) are hosts to a variety of diseases, including bacterial, viral, and parasitic. One infectious agent of particular importance in this species is pigeon paramyxovirus type 1 (PPMV-1) [1]. PPMV-1 is the primary cause of diseases in free-ranging and captive pigeons and doves, but viruses of the PPMV-1 genotype VI are known to infect other members of the family *Columbidae*, including wild birds and domestic poultry [2,3,4], thus posing a continuous threat to the poultry industry [5,6]. It is an antigenic and host variant of avian paramyxovirus Newcastle disease virus (NDV), which causes outbreaks with severe morbidity and mortality in chickens. Both PPMV-1 and NDV are enveloped viruses containing a negative-sense, single-stranded RNA genome that belongs to class II genotype VI (formerly known as genotype Vib or lineage 4b) of paramyxovirus type 1 [7,8,9]. These viruses are classified into the same serotype within the *Orthoavulavirus* genus, *Avulavirinae* subfamily, and *Paramyxoviridae* family within the order *Mononegavirales* [10,11].

In the late 1970s, PPMV-1 was predicted to have originated in the Middle East, which later spread to Europe [12], where the first outbreak was reported in pigeons in the early 1980s [13,14]. Since then, PPMV-1 has become endemic in many countries globally [15,16,17], largely attributed to long-distance migration, competition flights, ornamental exhibitions, and the trade of live birds [18]. Additionally, since 1981, pigeons in Egypt were reported to exhibit clinical signs comparable to NDV infection, and the presence of NDV antigens were detected in serum samples taken from diseased pigeons in 1984 [12,19]. 

The virulence of NDV strains in chickens is variable and classified into three pathotypes: lentogenic (non-virulent), mesogenic (intermediate virulent), and velogenic (highly virulent). These pathotypes are determined using assays, such as mean death time (MDT) of chicken embryos, intracerebral pathogenicity index (ICPI), intravenous pathogenicity index (IVPI), and genomic sequences analysis [20,21,22]. Additionally, the virulence phenotype in chickens is predicted based on the presence of multiple basic amino acids at the cleavage site motif of the viral fusion (F) protein [23,24,25,26,27]. However, most PPMV-1 strains are virulent in pigeons. NDV and PPMW-1 exhibit a range of differential clinical signs, including nervous (tremors of the neck and wings, bilateral or unilateral locomotor disorders, torticollis, paralysis, and disturbed equilibrium), digestive (polydipsia, polyuria, anorexia, and diarrhoea) [24,28,29], respiratory distress (gasping, coughing, sneezing, and tracheal rales), and death of up to 100% of the population [19].

PPMV-1 is enzootic in Egypt and causes significant negative economic impacts on commercial poultry production [4,30]. Proper and effective methods of prevention and control, in addition to diagnostic methods, are important for the protection of pigeons and other bird species from PPMV-1 [16]. While vaccination of racing pigeons against NDV is compulsory in many countries [31], most pigeon-rearing strategies in Egypt vary and do not necessitate routine immunisation, and they are, therefore, at greater risk of morbidity and mortality [30]. Thus, improving pigeon vaccination regimens against PPMV-1 could potentially reduce the disease burden in Egypt.

Several vaccination studies have evaluated the protective efficacy of available NDV poultry vaccines, such as live formulation of NDV LaSota or its variant Clone-30 strains, which belong to genotype II of class II paramyxovirus [32]. The immunogenicity of pigeon-derived genotype VI differs from the chicken-origin genotype II vaccine strain of NDV [33,34]; however, little is known about the protective properties of an inactivated PPMV-1 vaccine in comparison to the commercial NDV LaSota vaccine in pigeons [35]. Additionally, despite extensive research on the virus, further studies on the phenotypic characteristics of different strains of PPMV-1 prevalent in nature are needed. Experimental infections with contemporary strains dominating in the field could provide insights into their virulence phenotypes and assess the effectiveness of available commercial vaccines in mitigating their impacts.

This study, therefore, evaluates the phenotypic properties (MDT, ICPI, and IVPI) of the PPMV-1 strain isolated from pigeons (Pigeon/Egypt/Sharkia-19/2015/KX580988) in both chickens and pigeons. Additionally, we assess the effectiveness of available commercial paramyxovirus vaccines in protecting pigeons from this PPMV-1 isolate.

These findings will enhance our understanding, diagnosis, and prevention of PPMV-1 infections in regions with intensive pigeon rearing. They will also help in reducing the risk of disease transmission and optimising vaccination programs.

## 2. Materials and Methods

### 2.1. Ethics Considerations

The experiment design and protocol were approved by the Institutional Animal Care and Use Committee of Zagazig University, Egypt (approval ID ZU-IACUC/2/F/26/2020). This study was conducted in strict accordance with the approved guidelines for the Care and Use of Laboratory Animals. Birds were allowed to acclimatise to the facilities before the study began. Welfare checks were performed on the birds two to three times daily, and clinical scores were recorded to assess humane endpoints. Any bird showing signs of disease that met a humane endpoint was euthanised.

### 2.2. Birds

Domestic pigeons (*Colomba livia*; n = 99) used in this study were hatched and reared in a backyard rearing system for 4 weeks, non-vaccinated against NDV or PPMV-1. Before the experiment, the health status of the pigeons was observed for 7–14 days. Pigeons (n = 5) were randomly selected and euthanised for unchallenged and unvaccinated control histopathology. Blood samples and tracheal and cloacal swabs were collected from the remaining pigeons.

No pigeons expressed any clinical symptoms of disease, and pathological lesions were absent in the euthanised birds. The collected serum samples lacked avian paramyxovirus-1- (APMV-1) and avian influenza virus (AIV)-specific haemagglutination inhibition (HI) antibodies. The HI test was performed using positive-control hyperimmune serum against NDV, PPMV-1 [36], and H5 and H9 AIV (Department of Avian and Rabbit Medicine, Faculty of Veterinary Medicine, Zagazig University) according to the standard method of the World Organisation of Animal Health (WOAH) [37]. Antibody titres of 1:4 (16 haemagglutination units (HAU)) or greater were used to indicate past infection, current exposure, maternal antibodies, or vaccination titres. Likewise, swab samples were absent of APMV-1 or AIV pathogens, confirmed by propagation in SPF embryonated chicken eggs (SPF-ECEs) and identification by the haemagglutination (HA) assay.

One-day-old specific-pathogen-free (SPF) white leghorn chickens (n = 20; Koom Oshiem, Fayoum, Egypt) were used to study pathogenicity characterisation of the PPMV-1 strain and evaluate the vaccine’s efficacy. All birds were housed in separate experimental units at the Faculty of Veterinary Medicine, Zagazig University.

All animals, experimental procedures, and protocols were approved by the Intuitional Animal Care and Use Committee of Zagazig University, Egypt. The humane endpoints for chickens and pigeons inoculated with PPMV-1 were followed such that any infected bird showing signs of green watery diarrhoea, nervous symptoms (such as twisting of the head and neck or loss of balance), or difficulty in breathing (mouth breathing with an extended neck) was immediately euthanised and recorded as having reached severe disease signs.

### 2.3. Vaccines

Two vaccines were used in this study: (i) the live freeze-dried form of the LaSota lentogenic strain of NDV (CEVAC^®^ NEW L, Ceva Sante Animale Egypt, Al Sheikh Zayed, Giza), which contained 10^6^ EID_50_ of vaccinal virus/dose delivered via eye drops of 50 µL per dose, and (ii) formalised inactivated PPMV-1 (containing 10^10^ EID_50_/mL), obtained from the Veterinary Serum and Vaccine Research Institute (VSVRI; Abassia, Cairo, Egypt, Batch number 1907), which was prepared from the locally isolated Egyptian PPMV-1 (Pigeon/Egypt/Sharkia-19/2015/KX580988). The vaccine was delivered via subcutaneous injection in the centre of the back of the neck at a 0.5 mL dose per bird.

### 2.4. Pigeon Paramyxovirus-1 (PPMV-1) Isolate

The current study used the PPMV-1 strain Pigeon/Egypt/Sharkia-19/2015/KX580988 (GenBank ID: KX580988) [30], isolated from a native pigeon flock in Al-Shabanat, Sharkia, Egypt, during an outbreak in 2015. This flock was vaccinated with the NDV LaSota vaccine but suffered from noteworthy nervous symptoms and diarrhoea, with up to 80% mortality. Nephrosis, nephritis, enteritis, and a congested brain were also recorded during necropsy. This isolate was identified as PPMV-1 by using hyperimmune serum against PPMV-1 prepared by Hamouda et al. [36], with a haemagglutination inhibition (HI) titre of 4 log_2_. Subsequently, RT-PCR was used to identify the viral RNA, and sequencing analysis of the isolate confirmed the presence of a poly-basic fusion protein cleavage site, ^112^KRQKRF^117^, identifying the strain as velogenic PPMV-1 belonging to sub-genotype Vib.2, class II [30]. The isolate was propagated in the allantoic sac of 10-day-old SPF embryonated chicken eggs (SPF-ECEs) for 5 days of incubation at 37 °C and relative humidity of 70%. ECEs were checked daily for death, and dead embryos were chilled overnight upon identification. Post-live and dead embryos were investigated, and allantoic fluids (Afs) were harvested. Collected Afs were tested for virus titres by a microplate haemagglutination (HA) test using 1% (*v*/*v*) washed chicken red blood cells (RBCs), performed as described by the WOAH [37]. 

### 2.5. Pathogenicity Characterisation

The pathogenic potential of the isolate was determined by the mean death time (MDT), intracerebral pathogenicity index (ICPI), and intravenous pathogenicity index (IVPI) tests.

#### 2.5.1. Mean Death Time (MDT) and Virus Titration

The MDT test was conducted using 10-day-old SPF-ECEs and calculated according to the method previously described [7]. The value of MDT was determined as the mean time in hours (h) necessary for the death of all ECEs. Specifically, MDT values greater than 90 h indicate low virulence, between 60 and 90 h indicate moderate virulence, and less than 60 h indicate high virulence. The embryonic infectious dose of 50% (EID_50_) of the isolate was calculated using the Reed and Muench method [38] in SPF-ECEs. The virus stock was diluted in phosphate-buffered saline and standardised to 10^6^ EID_50_/0.1 mL.

#### 2.5.2. Intracerebral Pathogenicity Index (ICPI) and Intravenous Pathogenicity Index (IVPI)

The 5-week-old domestic pigeons (*Colomba livia*; n = 30) and 1-day-old SPF chickens (n = 20) were used to determine and compare ICPI [37,39] and IVPI [7] values between the natural pigeon host and the chicken spillover host.

**Figure 1:**
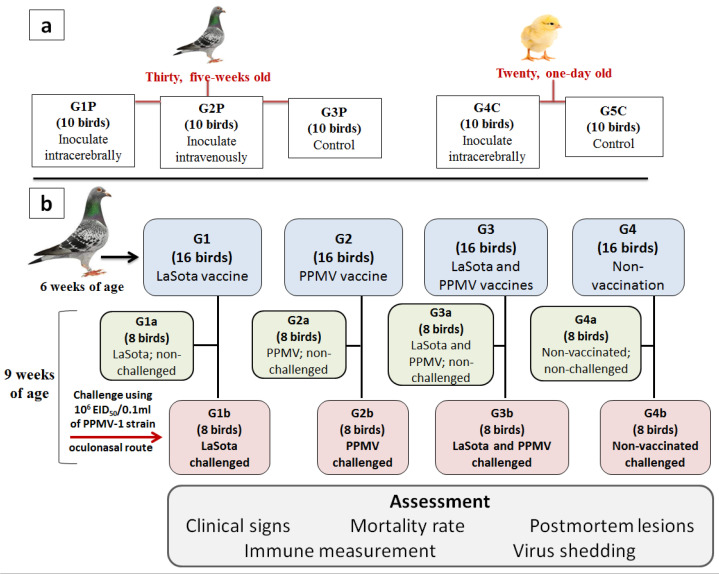
Summary of the experimental design: (**a**) Design of pathogenicity characterisation. (**b**) Design of vaccines’ assessment against those challenged with a field strain of PPMV-1, “Pigeon/Egypt/Sharkia-19/2015/KX580988”. G1a, G2a, G3a, and G4a were kept as control groups to monitor any natural infection exposure from the environment to these pigeons. G1b, G2b, G3b, and G4b were challenged with the PPMV-1 strain.

Three groups (G1P–G3P) of pigeons (n = 10/group) and two groups (G4C–G5C) of SPF chickens (n = 10/group) were prepared for inoculation. Groups G1P and G4C were inoculated intracerebrally (I/C) with 50 µL of the ten-fold dilution of the fresh virus Af stock containing HA titre of 2^10^ (10^7.6^ EID_50_) in 0.1 mL. Group G2P was inoculated intravenously (I/V) with 0.1 mL of the ten-fold dilution of the same fresh virus Af stock (Figure 1a). Groups G3P and G5C were used as mock-inoculated control birds and were housed separately from the other birds. The birds were observed and examined daily for 8 and 10 days in intracerebrally and intravenously inoculated groups, respectively. The observed clinical signs and mortalities were recorded and scored to determine the pathogenicity indexes. 

The ICPI involves scoring sick or dead (0 = normal, 1 = sick, and 2 = dead). ICPI values below 0.7 are considered low virulence, and values greater than 0.7 are virulent [37,40]. The IVPI involves scoring illness (0 = normal, 1 = sick, 2 = paralysed or nervous signs, and 3 = dead) after IV inoculation. Velogenic strains have an IVPI score of 2–3, mesogenic of 0–0.5, and lentogenic of 0 [39].

### 2.6. Evaluating Vaccine Efficacy

#### 2.6.1. Pigeon Experiment Design

The 6-week-old clinically healthy domestic pigeons (n = 64) were divided into 4 groups (G1–G4) of 16 birds each. The experimental pigeons were kept in the experiment units under controlled conditions for the period of the study and received consistent feed and water ad libitum. Groups G1, G2, and G3 were vaccinated with live NDV LaSota, inactivated PPMV, and a dual vaccine of live NDV LaSota and inactivated PPMV, respectively (Figure 1b). The NDV LaSota vaccine was applied on the first day of the experiment and boosted after two weeks (eight weeks of age). On the second day of the experiment, the formalised-inactivated PPMV vaccine was administered once. G4 was kept as the control (non-vaccinated) group.

At nine weeks of age, the four pigeon groups were subdivided into eight subgroups (G1a, G2a, G3a, and G4a, and G1b, G2b, G3b, and G4b). The G1b, G2b, G3b, and G4b subgroups were challenged intra-oculonasally with a dose of 0.1 mL, containing 10^6^ EID_50_ of the challenge virus “Pigeon/Egypt/Sharkia-19/2015/KX580988”. The G1a, G2a, G3a, and G4a subgroups remained unchallenged. Serum samples were collected weekly from each subgroup (3 birds per subgroup) to determine the avian paramyxovirus-specific inhibitory antibody titres by HI. Post-challenge, pigeons were monitored twice daily for 14 days to record clinical signs and mortality. Clinical signs were scored according to their clinical condition (0 = healthy; 1 = diseased; 2 = nervous signs; 3 = dead). The clinical index was calculated analogously to determining the IVPI. As determined by the IVPI, the course of disease from inoculation was nearly the same as that from natural infection via the oculonasal route. In addition, the observation period was extended to 14 days to monitor recovery. Pigeons that died suddenly during the experiment were immediately necropsied. Birds that were culled upon reaching humane endpoints, as well as those that recovered from infection and did not experience humane endpoints, were euthanised at the end of the observation period for post-mortem examination and tissue collection to detect disease lesions. Three pooled tracheal and cloacal swabs were collected separately from six randomly selected pigeons (two swabs per pool) at three, five, and seven days post-challenge (dpc) to determine the virus shedding.

#### 2.6.2. Immune Response by Haemagglutination Inhibition (HI)

The HI test was performed on the collected serum samples according to the procedures listed in the WOAH [39] using 4 haemagglutination units (HAU) of the PPMV-1 strain, the LaSota vaccine strain of NDV (Pestikal^®^ LASOTA SPF), and 1% (*v*/*v*) RBCs. The serum samples were thermally inactivated for 30 min at 56 °C. Two-fold serial dilutions were carried out, and the HI titre was expressed as log_2_ of the reciprocal of the highest serum dilution that suppressed agglutination of RBCs.

#### 2.6.3. Virus Shedding Determination

As previously mentioned, tracheal and cloacal swabs were collected on the 3rd, 5th, and 7th dpc from different subgroups. These swabs were suspended in 1.5 mL of minimum essential media (MEM) containing antibiotics (Penstrept, Lonza) then clarified by centrifugation at 3000 revolutions per minute (rpm) for 10 min at 4 °C. Virus shedding was quantified and implemented using real-time quantitative reverse transcription polymerase chain reaction (RT-qPCR). Viral RNA was extracted from the supernatant using the QIAamp MinElute Virus Spin kit (QiagenGmbH, Hilden, Germany), according to the manufacturer’s protocol. The extracted RNA was subjected to one-step real-time RT-PCR using the AgPath-ID^TM^ one-step RT-PCR Kit (Applied Biosystems, Thermo Fisher Scientific Inc., Waltham, MA, USA) for detection and titration of PPMV-1. The following primers and probe specific to the F protein gene, as designed by Sabra et al. [41], were used: forward primer 5′-TGATTCCATCCGCAGGATACAAG-3′, reverse primer 5′- GCTGCTGTTATCTGTGCCGA-3′, and probe F-4876 5′-[6-FAM] AAGCGYTTCTGTCTCYTTCCTCCT [BHQ_1]–3′. The amplification was performed in an Applied Biosystems™ StepOne™ Real-Time PCR System (Thermo Fisher Scientific Inc.) with cycle conditions, as mentioned by Fuller et al. [42]. Overall, the amount of viral RNA in the swab samples was based on the threshold cycle (Ct) values obtained by RT-qPCR.

### 2.7. Microscopic Examination for Pathological Dissemination of PPMV-1 in Non-Vaccinated/Challenged Pigeons

#### 2.7.1. Histopathology

In the non-vaccinated/challenged group (G4b), tissue samples were collected from different organs (brain, trachea, lung, heart, liver, pancreas, spleen, proventriculus, intestine, and kidneys) from the freshly dead and euthanised symptomatic pigeons. The collected tissues were fixed in 10% neutral-buffered formalin before being embedded in paraffin and sectioned in duplicate to a 3 µm thickness. For histopathology, slides were stained by haematoxylin and eosin (H&E) stain and examined by a light microscope [43].

#### 2.7.2. Immunohistochemistry

To produce hyperimmune serum against PPMV-1, rabbits were administered a series of injections by the schedule outlined by Samiullah et al. [44]. MagneProtein G Beads for Antibody Purification (Promega Corporation, Madison, WI, USA) were used to purify antibodies following the manufacturer’s instructions.

Tissue sections were placed on slides coated with poly-L-lysine, which were then deparaffinised and rehydrated. Heat-induced antigen retrieval was performed using a scientific microwave at 98 °C for 20 min, and blocking of non-specific protein binding and endogenous peroxide application were performed. Tissue sections were incubated overnight at room temperature with a monoclonal primary antibody (rabbit anti-PPMV-1 immunoglobulin (Ig)), followed by incubation with horseradish peroxidase-conjugated goat polyclonal secondary antibody to rabbit Ig (SM802 EnVision^TM^ FLEX/HRP). Colour was developed with a 3,3’-diaminobenzidine (DAB) substrate (DM827 EnVision^TM^ FLEX DAB þ chromogen) [45]. Positive results appeared as a brown precipitate localised at the site of binding and were observed under an optical microscope. Negative tissue slides were established by adding negative serum instead of the primary antibody on non-vaccinated/challenged pigeon (G4b) tissue specimens and used as a reaction guide. Additionally, tissue specimens of non-vaccinated, non-challenged pigeons (G4a) were incubated with the primary anti-PPMV-1 antibodies.

### 2.8. Statistical Analysis

Several linear and generalised linear models were performed, and the Akaike Information Criterion (AIC) was used to select the model with the best quality to fit the data. A generalised linear model (GLM) with a gamma distribution and log link function was employed to analyse HI titres, as it had the lowest AIC. The HI titres are continuous, positively skewed data, and the gamma distribution is well suited for modelling such data. The log link function ensures that predicted values remain positive on the original HI titre scale.

The generalised linear model gamma regression with the log link function was used to assess the pattern of the HI titre change using two different antigens within five groups over the subsequent five weeks of the experiment. The probability function of the gamma distribution is as follows:

$$P\left(y;\;\alpha ,\beta \right)=\frac{y^{\alpha -1}exp(-y/\beta )}{\Gamma (\alpha )\beta^{\alpha }}$$

For y (HI titre) > 0, α > 0 (the shape parameter), and β > 0 (the scale or the spread parameter), where E[y] = αβ and var[y] = αβ2. Note that Γ () is the gamma function. The y variable is then changed to z, as: y → z = exp{y}, to receive the density of the log-gamma distributed random variable, Z = exp{Y} [46]. 

The analysis was conducted in R (version 2023.06.1) using the “glm” function. Maximum likelihood estimation was used to fit the model. Goodness-of-fit was evaluated using chi-square tests and residual deviance. Estimated coefficients and their *p*-values were reported to assess the significance of predictor variables on the log-transformed mean change in HI titres. Probability (*p*) values < 0.05 were considered significant. The model coefficients were exponentiated to provide insights into the actual effects of the predictors (antigen type, treatment groups, and weeks of experiment) on the HI titre change.

## 3. Results

### 3.1. The PPMV-1 Strain Caused Severe Disease Symptoms and Was Lethal to Chicken Embryos 

Inoculated SPF-ECEs showed severe congestion of visible veins, along with subcutaneous haemorrhages, and stunted growth in the first passage in 80% of inoculated embryos at 96 h, which increased to 100% within 48 to 72 h post-inoculation (hpi) in the third passage. The embryo death reached 100% of inoculated embryos within 72 hpi.

### 3.2. The PPMV-1 Strain Was Virulent and Lethal to Chickens and Pigeons

**Figure 2:**
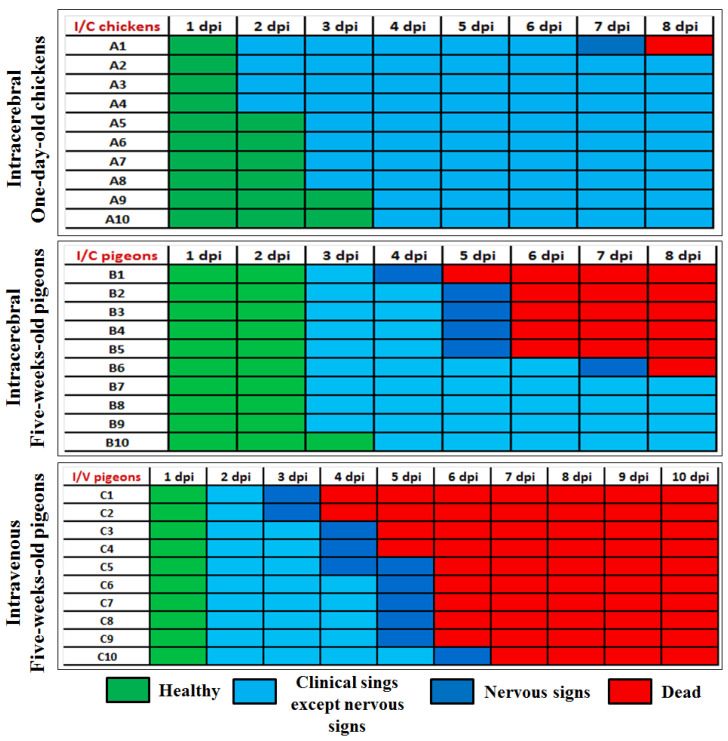
Virulence (ICPI and IVPI) assessment of the PPMV-1 strain (Pigeon/Egypt/Sharkia-19/2015/KX580988) in pigeons and chickens. The birds were inoculated via intracerebral (I/C) and intravenous (I/V) routes to 1-day-old chickens and 5-week-old pigeons. The clinical scores were recorded until 10 days post-infection (dpi). The clinical scores are represented as healthy (green), displaying variable clinical signs, such as ruffled feathers, depression, anorexia, and greenish diarrhoea (light blue, except nervous signs in dark blue), and dead (red). A1–A10 are numbers of chickens in the I/C group (G4C), B1–B10 are numbers of pigeons in the I/C group (G1P), and C1–C10 are numbers of pigeons in the I/V group (G2P).

The virulence of the PPMV-1 strain in pigeons and chickens was determined by adopting standard MDT, ICPI, and IVPI protocols. The MDT was 86.4 ± 5.88 h in SPF-ECEs, suggesting that the virulence of this isolate was moderate. The observed ICPI in 1-day-old SPF-infected chickens was 0.8, while it was 0.96 in 5-week-old infected pigeons. This supports the classification of this PPMV-1 strain as a virulent pathotype; thus, outbreaks with closely related strains come under the notifiable outbreaks to the WOAH. The observed IVPI score in 5-week-old pigeons was found to be 2.11, which deemed the strain velogenic. The presence of a multi-basic cleavage site motif in the virus F protein, together with in ovo and in vivo infection scores determined via MDT, ICPI, and IVPI assays (Figure 2), confirmed that the strain is a virulent pathotype for both pigeons and chickens. 

### 3.3. Pigeons Vaccinated with PPMV or a Dual Vaccine (NDV LaSota and PPMV) Showed Protection from Clinical Disease and Mortality When Challenged with PPMV-1

**Figure 3:**
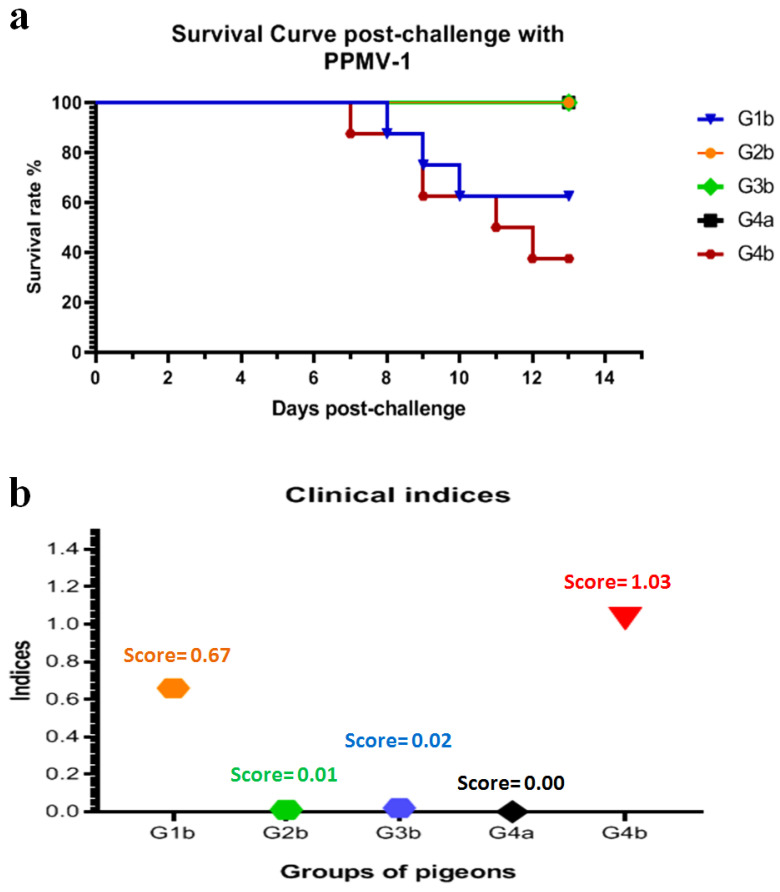
Survival rates and clinical indices post-challenge with PPMV-1. (**a**) Survival curves of vaccinated pigeons with the tested vaccines post-challenge. (**b**) Clinical indices of vaccinated pigeons with the tested vaccines post-challenge. G1b: vaccinated with NDV LaSota/challenged with PPMV-1; G2b: vaccinated with PPMV/challenged with PPMV-1; G3b: dual-vaccinated with LaSota and PPMV/challenged with PPMV-1; G4a: non-vaccinated/non-challenged; G4b: non-vaccinated/challenged.

**Figure 4:**
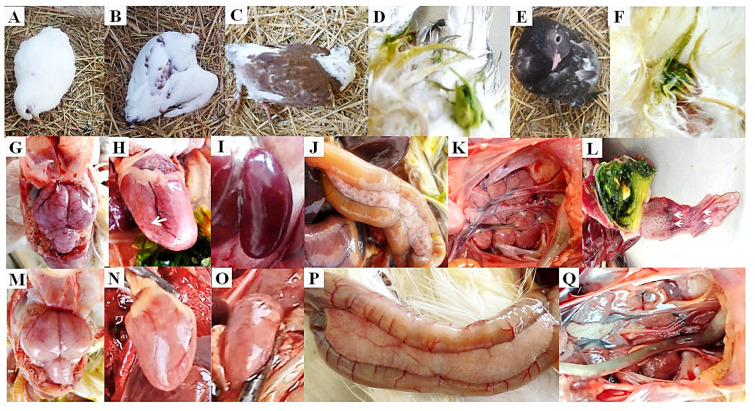
Clinical and post-mortem findings of pigeons vaccinated/challenged with PPMV-1 strain. (**A**) Torticollis in non-vaccinated/challenged pigeon (G4b). (**B**) Wing paralysis in non-vaccinated/challenged pigeon (G4b). (**C**) Complete paralysis in non-vaccinated/challenged pigeon (G4b). (**D**) Severe greenish diarrhoea in non-vaccinated/challenged pigeon (G4b). (**E**) Opisthotonos position in pigeon of NDV LaSota vaccinated/challenged (G1b). (**F**) Greenish diarrhoea in pigeon of NDV LaSota vaccinated/challenged (G1b). (**G**) Severe congested and haemorrhagic brain in non-vaccinated/challenged pigeon (G4b). (**H**) Congested heart with minute petechial haemorrhages in non-vaccinated/challenged pigeon (G4b). (**I**) Enlarged and severe congested spleen with haemorrhages in non-vaccinated/challenged pigeon (G4b). (**J**) Pancreatitis with punctate haemorrhages in non-vaccinated/challenged pigeon (G4b). (**K**) Severe nephrosis in non-vaccinated/challenged pigeon (G4b). (**L**) Haemorrhages at junction between oesophagus and proventriculus with greenish content in the gizzard of non-vaccinated/challenged pigeon (G4b). (**M**) Moderate congestion in brain of pigeon in NDV LaSota vaccinated/challenged group (G1b). (**N**) Mild congestion (apparently normal) of heart in pigeon of NDV LaSota vaccinated/challenged group (G1b). (**O**) Enlarged spleen with few haemorrhages in pigeon of NDV LaSota vaccinated/challenged group (G1b). (**P**) Pancreatitis with necrosis in pigeon of NDV LaSota vaccinated/challenged group (G1b). (**Q**) Moderate nephrosis in pigeon of NDV LaSota group (G1b).

Survival curves post-challenge in vaccinated and unvaccinated pigeons using the tested vaccines are shown in Figure 3a. The clinical signs of PPMV-1 infection in the non-vaccinated/challenged group (G4b) were observed at 5 dpi. All infected birds (8/8 (100%)) suffered from ruffled feathers, lethargy, and anorexia, one bird showed respiratory signs, and greenish diarrhoea was seen in 5 birds at 7 dpi. At 8 dpi, 6/8 (75%) birds showed signs of head tremors, torticollis, opisthotonos position, and wing or complete paralysis (Table 1 and Figure 4A–D). All birds experienced moderate to severe disease signs, and the cumulative clinical score was 1.03 (Figure 3b). Between 7 and 12 dpi, 5/8 (62.5%) birds died, and the remaining 3 (37.5%) pigeons expressed clinical signs but below the humane endpoint threshold.

Pigeons infected with PPMV-1 after vaccination with either PPMV alone (G2b) or a dual vaccine (NDV LaSota and PPMV; G3b) showed no apparent clinical disease signs and remained nearly normal, with only 1/8 (12.5%) per group displaying mild diarrhoea (G2b) and head tremors (G3b) at 13 days post-challenge (dpc), which later recovered. Thus, the vaccine protection efficacy from mortality was 100%. The pigeons in the NDV LaSota vaccinated/PPMV-1 challenged group (G1b) showed relatively reduced protection from clinical disease signs compared to those that received inactivated PPMV alone or the dual (NDV LaSota and PPMV) vaccine. Clinical manifestations in these pigeons began at 7 dpc, and the overall clinical score remained at 0.67, with a survival rate of 62.5% (Figure 3b). This was relatively less pronounced in terms of severity and frequency compared to the non-vaccinated/PPMV-1 challenged group (G4b; Table 1 and Figure 4E,F). The vaccinated/non-challenged and non-vaccinated/non-challenged groups (G1a–G4a; to monitor natural exposure from the environment) showed no clinical disease signs or mortality, confirming that the experimental birds were not exposed to PPMV-1 or any other natural infections commonly causing clinical disease in pigeons.

### 3.4. Pigeons Vaccinated with PPMV or Dual Vaccines (NDV LaSota and PPMV) Showed Low Severity of Post-Mortem Lesions after PPMV-1 Challenge

The severity of disease induced by virus challenge in vaccinated versus non-vaccinated pigeons was assessed by observing post-mortem lesions. Following the virus challenge, all pigeons (n = 8) in the non-vaccinated control group (G4b) either died or were culled because of reaching the humane endpoint. The severity of gross lesions in these birds was apparent, including congestion of all visceral organs (septicaemia), a congested and haemorrhagic brain, and pancreas with punctate haemorrhages or necrosis. The spleen was enlarged, congested (n = 4), and atrophied (n = 1). Seven kidneys displayed nephrosis (n = 5) or a nephritis-like condition (n = 2). Haemorrhages were observed between the oesophagus and proventriculus and enteritis with greenish content in the gizzard and intestine. Further, 2/8 (25%) pigeons presented minute petechial haemorrhages in the heart. The thymus and bursa of Fabricius were atrophied in 5/8 (62.5%) and 4/8 (50%) of the infected pigeons, respectively (Figure 4G–L and Table 2). In the group vaccinated with NDV LaSota (G1b), 5/8 (62.5%) pigeons either died or were euthanised after reaching humane endpoints. These birds exhibited post-mortem lesions with moderate severity (Figure 4M–Q and Table 2). In contrast, in the groups vaccinated with PPMV (G2b) or dual-vaccinated with both LaSota and PPMV (G3b), only 1/8 (12.5%) pigeons from each group reached the humane endpoint. Post-mortem analysis showed relatively mild, less pronounced gross lesions in their visceral organs (Table 2).

### 3.5. PPMV-1 Induced Extensive Microscopic Lesions in Visceral Organs of Non-Vaccinated/Challenged Pigeons

**Figure 5:**
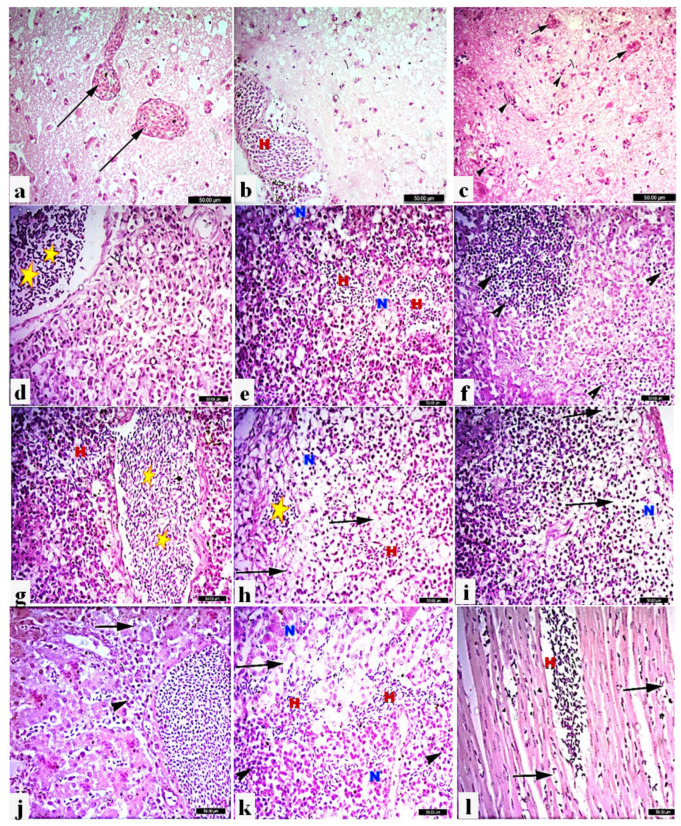
Histopathological lesions in different tissue organs of non-vaccinated infected pigeons with PPMV-1. H&E X400, bar indicates 50 μm. Brain: (**a**) Congestion of brain capillaries (long arrows). (**b**) Sub-meningeal haemorrhages (H). (**c**) Multifocal degeneration and swelling of neurons gliosis (arrow heads) and degenerated neurons (short arrows). Pancreas: (**d**) Severe congestion (stars). (**e**) Multifocal haemorrhages (H) with degeneration and necrosis of pancreatic acini (N). (**f**) Multifocal aggregations of lymphocytic infiltrations (arrowhead). Spleen: (**g**) Severe congestion of sinusoids and splenic blood vessels (stars), with multifocal haemorrhages (H). (**h**) Multifocal haemorrhages (H) and severe depletion of subcapsular lymphoid follicles (long arrows), with necrosis (N), and congested blood vessels (star). (**i**) Severe depletion of subcapsular lymphoid follicles (long arrows), and necrosis (N). (**j**) Liver: severe congestion, focal degeneration (long arrow), and discrete necrosis of some hepatic cells, along with mild leukocytes around bile duct (arrowhead). (**k**) Kidneys: intratubular haemorrhages (H), degeneration of renal tubular epithelium (long arrow), and focal necrosis (N) with leukocyte infiltration (arrowhead). (**l**) Heart: intermuscular congestion (long arrows) and haemorrhages (H).

Microscopic lesions in the brain, pancreas, spleen, liver, kidneys, and heart were examined after H&E staining. Histopathological pictures of the brain revealed congested capillaries, sub-meningeal haemorrhages, and focal cortical haemorrhages. Multifocal degeneration and swelling of neurons were also observed, along with focal gliosis (Figure 5a–c). The pancreas of infected pigeons showed necrotic pancreatitis characterised by severe congestion, multifocal haemorrhage, and multifocal aggregations of lymphocytic infiltrations, as well as degeneration and necrosis of pancreatic acini (Figure 5d–f). Multifocal haemorrhages, congestion of sinusoids and splenic blood vessels, and lymphoid depletion of the white pulp of the spleen were observed (Figure 5g–i). Microscopic examination of livers showed severe congestion, focal degeneration, and discrete necrosis of some hepatic cells, along with mild leukocytes around bile ducts (Figure 5j). The kidney displayed tubulointerstitial nephritis characterised by intratubular haemorrhages, degeneration of renal tubular epithelium, and focal necrosis with leukocytic infiltrations (Figure 5k). Intermuscular congestion and haemorrhages were found in the heart (Figure 5l). By contrast, no histopathological changes were observed in respective tissues of the non-vaccinated/non-challenged pigeons (Supplementary Figure S1).

### 3.6. PPMV-1 Expressed Strong Immunohistochemistry Signals in Non-Vaccinated/Challenged Pigeons

**Figure 6:**
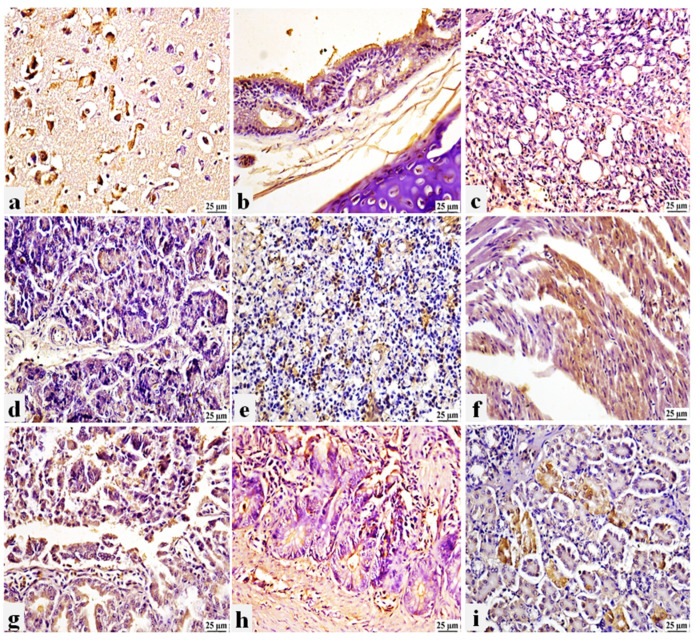
Photomicrograph of immunohistochemistry (IHC) of PPMV-1 presence in different tissue organs of non-vaccinated/challenged pigeons. (**a**) Brain: positive expression in degenerated neurons. (**b**) Trachea: positive expression in the tracheal epithelium and in the inflammatory cells of the propria submucosa. (**c**) Lung: positive expression in air capillaries. (**d**) Pancreas: positive expression in the exocrine epithelial cells. (**e**) Spleen: positive expression in the splenic parenchyma. (**f**) Heart: positive expression in the cardiac muscle. (**g**) Proventriculus: positive expression in the glandular epithelium and desquamated epithelial cells. (**h**) Intestine: positive expression in intestinal mucosa. (**i**) Kidneys: positive expression in the renal tubular epithelium.

The PPMV-1 antigen was found to be expressed in various tissue organs of pigeons, where a strong positive peroxidase reaction was detected. This reaction was shown in the brain neurons (Figure 6a), tracheal epithelium, with the inflammatory cells infiltrated in the propria submucosa (Figure 6b), and air capillaries of the lung (Figure 6c). The exocrine pancreatic acini (Figure 6d), splenic parenchyma (Figure 6e), and cardiomyocytes of some affected pigeons (Figure 6f) had viral antigen expression. Furthermore, the acinar epithelium of the proventriculus with the desquamation epithelial cells (Figure 6g), the intestinal mucosa (Figure 6h), and the renal tubular epithelium (Figure 6i), with the inflammatory cells occupying the interstitial tissue, showed positive immunolabeling. This suggests that PPMV-1 is systemically distributed in internal organs with clear neuro-viscero tropism. Negative immune staining was observed in the non-vaccinated/non-challenged pigeons (G4a) using the prepared specific anti-PPMV-1 antibody. Similarly, negative immunostaining was detected in non-vaccinated/challenged pigeons (G4b) following staining with negative serum (Supplementary Figure S2).

### 3.7. Vaccination with PPMV Alone or in Combination with NDV LaSota Produced Predicted Protective Levels of HI Antibody Titres in Pigeons 

Serum was collected from vaccinated pigeons and subjected to HI assays. At three weeks post-vaccination, pigeons vaccinated with PPMV and challenged (G2b) had an HI titre (log_2_) of 6 ± 0.47 and 5.67 ± 0.45 to LaSota and PPMV-1 antigens, respectively. Pigeons dual-vaccinated with LaSota and PPMV and challenged (G3b) had an HI titre of 5 ± 0.39 to both antigens of LaSota and PPMV-1 (Table 3).

**Figure 7:**
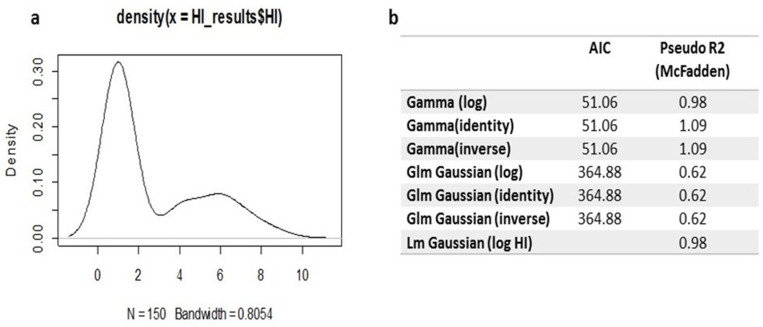
(**a**) Density plot showing the positive skewness in the HI titre distribution. (**b**) Model selection criteria for linear and generalised linear designs based on the Akaike Information Criterion (AIC). N = number of collected serum samples from all experiment pigeon groups throughout the experiment periods.

Initially, plotting the density distribution of the HI titre revealed a positive skewness in the data (Figure 7a). Therefore, the log of the HI titre using the gamma distribution was recommended to represent the model with the data, which was selected as the best fit after comparing other routinely used models, such as linear, general linear, and generalised linear models. A comparison to the linear or generalised linear models revealed that the generalised linear model with gamma distribution and the log link function was the best, having the lowest AIC (Figure 7b). The predicted values of the generalised linear gamma model (GLGM) for the mean antibody titres using HI clarified that there were no differences between the use of LaSota (as a heterologous antigen) and PPMV-1 (as a homologous antigen), as shown in Figure S3.

The model coefficients (Figure S3) and estimated marginal means (Table 3) indicated that the antibody (Ab) titres were significantly developed at three weeks post-vaccination (the time of challenge) in groups vaccinated with inactivated PPMV (G2b; 5.67 ± 0.45:6 ± 0.47) and NDV LaSota with inactivated PPMV (G3b; 5 ± 0.39), compared to the NDV LaSota group (G1b) and unvaccinated groups (G4a and G4b). The Ab levels of the same groups (G2b and G3b) continuously increased post-challenge and reached the highest titres at five weeks post-vaccination (Figure S3), particularly in G2b, either using PPMV or NDV LaSota antigens, with a coefficient of 1.56. This indicated that this group at week 5 post-vaccination was related to a highly significant (56 times) increase in HI titres, with a *p*-value equal to 2.2 × 10^−16^, compared to other groups and weeks. 

In the NDV LaSota vaccinated group (G1b), the development of HI Abs was slow and not detected until the 5th week (Figure S3). Although its Abs level was high (coefficient value = 1.19 in the 5th week), it was lower than from the time of the PPMV-1 challenge, as shown in Figure S3, and the increase in HI titres at the 5th week was not significant (*p* = 0.06).

### 3.8. The Inactivated PPMV Vaccine and NDV LaSota with PPMV Dual Vaccine Reduced Virus-Shedding Pigeons Challenged with PPMV-1

The vaccinated group with the inactivated PPMV vaccine (G2b) inhibited virus shedding. The group that received the live NDV LaSota with inactivated PPMV vaccine (G3b) prevented the tracheal shedding and markedly minimised the cloacal shedding, with a Ct value of 37 in 1/3 vial swabs at 7 dpc. In the live NDV LaSota vaccination group (G1b), virus shedding was detected in both tracheal and cloacal swabs as early as day five post-challenge; however, it reduced the number of birds that shed the virus, contrasting that in the unvaccinated challenged group (G4b; Table 4).

## 4. Discussion

PPMV-1 is now a serious global hazard to bird populations, with pigeons being particularly vulnerable [47,48]. The strain “Pigeon/Egypt/Sharkia-19/2015/KX580988”, previously isolated from a vaccinated native pigeon flock in Egypt, was classified as a velogenic strain that belonged to sub-genotype VIb.2, class II. This study was designated to determine the virus pathotype characteristics in pigeons and chickens and to evaluate the protective efficacy of available commercial avian paramyxovirus vaccines against the velogenic strain of PPMV-1. Based on the observed MDT value (86.4 ± 5.88 in 10-day-old SPF-ECEs), ICPI value (0.8 and 0.96 in 1-day-old SPF-chickens and 5-week-old pigeons, respectively), and IVPI scores (2.11), the Pigeon/Egypt/Sharkia-19/2015/KX580988 strain exhibited velogenic pathotype characteristics. Earlier reported studies displayed similar pathogenicity for PPMV-1 using MDT and ICPI in chickens [49,50,51,52,53,54], although the poly-basic fusion protein in the cleavage site identified this strain as velogenic PPMV-1 [30]. The majority of PPMV-1 strains are mesogenic or lentogenic for chickens, according to ICPI and MDT tests, even though they have been demonstrated to have deduced amino acid motifs, suggesting high pathogenicity [25,55]. These imply that other factors affect the pathogenic phenotype of these viruses, and that the cleavage site is not the only factor regulating PPMV-1’s pathogenicity in various species [26,33]. According to the WOAH standards, the ICPI of the strain used in the present study belongs to the virulent or velogenic category [37]. Notably, the ICPI (0.96) was relatively high in pigeons, with more pigeons showing nervous signs (n = 4/10 (40%)) and mortality (n = 6/10 (60%)) than chickens. Considering that pigeons are the natural host of the PPMV-1, it may not be suitable for its assessment to be carried out in chickens only [35]. However, when the PPMV-1 strain was tested for pathogenicity and clinical symptoms after successive passages in chickens, it became more virulent [1,16,26]. This pilot study and others [35] concluded that to evaluate the real virulence of PPMV-1, pigeons should be employed for pathogenicity testing.

Interestingly, the IVPI value indicated that the PPMV-1 was velogenic, with a score of 2.11 in pigeons, accompanied by mortality (n = 10/10 (100%)) within 7 days post-inoculation. The different degrees of virus pathogenicity in pigeons are related to different inoculation routes (intravenous or intracerebral). In the intravenous route, the virus enters the bloodstream immediately and subsequently spreads to different organs at a faster rate [56]. This systemic route of the inoculated virus is the cause of nervous involvement [57], and the high mortality that reaches 100%. According to the bioassay results in this prospective study, the PPMV-1 strain showed pathogenicity in both chickens and pigeons.

Considering the increase in PPMV-1 infections in recent years, disease prevention is required. Effective vaccinations or other preventative and control measures need to be developed and applied in pigeon lofts to reduce losses and the risk of disease transmission to other birds [58]. 

Consistent with previous studies [51,53,54,59], the non-vaccinated/challenged pigeons exhibited typical clinical signs and gross lesions. It is important to note that the nervous signs and greenish diarrhoea were the common signs, as documented by Śmietanka et al. [1], Zhan et al. [53], and Shalaby et al. [60], and they were accompanied by lesions recorded in the brain and digestive tract in 100% of pigeons. Primarily through the pattern of noticed signs and lesions, it is expected that this PPMV-1 strain has neurotropic and viscerotropic potentiality. Moreover, Chang et al. [51] concluded that virus titres were higher in the brain, intestine, and lung than in other tissues. 

Clinical signs within this experiment were observed at five dpi, coinciding with that previously recorded by Xie et al. [34] and Chang et al. [51], yet contradicting other studies with clinical signs observed as early as two to four dpi [53,54,59]. This variation in incubation periods may be the result of virus properties or virulence, the dose in the inoculum, and host features, such as age, breed, and immune response [1]. Additionally, the clinical score recorded by the infected pigeons reached 1.03, and the morbidity and mortality rates were 100% and 62.5%, respectively. 

PPMV-1 was able to cause systemic infection, which had a wide range of tissue distribution in pigeons, along with the prominent neurological and intestinal symptoms, and was broadly consistent with pronounced lesions in these organs. This suggests that PPMV-1 is a systemic virus with neuro- and viscero-tropism. 

The microscopic lesions observed in multiple organs of PPMV-1-infected pigeons were similar to those noted in previous reports [35,59,60,61,62]. Among the common gross lesions and microscopic changes that caught our attention were those in the brain, informing sub-meningeal and focal cortical haemorrhages, multifocal degeneration, and swelling of neurons with focal gliosis. The central nervous system (CNS) involvement in PPMV-1 disease was prevalent, particularly given the neurologic clinical signs often observed. This indicates the tropism of the PPMV-1 strain for the nervous tissues. Necrotic pancreatitis was most often observed in pigeons and is considered an acute change that might cause animal death due to systemic inflammatory response syndrome and multiple organ failure as a result of releasing activated proteolytic enzymes and pro-inflammatory cytokines into the bloodstream [63]. Tubulointerstitial nephritis was also observed, which could contribute to the loss of homeostasis [61]. Lymphoid depletion of the spleen’s white pulp was recorded in the presence of viral antigen expression in the IHC, which suggests that PPMV-1 may cause lymphoid depletion. However, only a few studies had reliable confirmation of lymphocytosis [61]. On the other hand, notable alterations in lymphoid organs have not always been described in PPMV-1 outbreaks [64,65]. Although IHC analysis has been adopted a few times in prior studies [60,61], it confirmed the incrimination of PPMV-1 responsibility for changes in the collected tissue organs by detecting strong PPMV-1 antigen expression. This suggests that the use of IHC to detect PPMV-1 infections in tissues could be a potential tool to trace the viral pathway in tissues and confirm its lesions.

All the vaccinated groups had a lower severity and frequency signs of disease compared to the non-vaccinated pigeons. Importantly, the pigeons had absence of disease against the PPMV-1 challenge when vaccinated with the inactivated PPMV vaccine alone or in combination with NDV LaSota. The clinical scores for both vaccination programs were 0.01 and 0.02, respectively, and their corresponding morbidity rates were both 12.5%. On the other hand, the NDV LaSota vaccination had a lower protection rate, with a clinical score of 0.67 and a 62.5% morbidity rate. Both vaccine programs containing inactivated PPMV protected 100% of pigeons against mortality, while the LaSota vaccine protected 62.5%. This indicated that the inactivated PPMV vaccine provided good protection. Similar observations were reported by Zhang et al. [35], Amer et al. [58], Stone [66], and Hassan [67], who conducted their investigations on the inactivated PPMV vaccine either locally and commercially [58,67] or prepared from an isolated strain [35,66] and NDV-II vaccines against PPMV-1 challenge in pigeons. They recommended that the homologous vaccine is more efficacious than the NDV-II vaccines, such as Ulster, LaSota, and Hitchner B1, and the protection rate reached 100% against morbidity and mortality. Serologically, the vaccination programs containing inactivated PPMV-1, whether alone or with NDV LaSota, developed significantly higher inhibitory antibody levels (by HI assay) in pigeons from three weeks post-vaccination (initial PPMV-1 challenge) than the NDV LaSota vaccine alone. Inhibitory antibody levels also increased over time post-challenge, suggesting that the PPMV-1-matched vaccine could induce an elevated humoral response. This finding mirrors the results of Zhang et al. [35]. 

NDV LaSota and PPMV-1, as diagnostic antigens against HI antibodies of PPMV-1, showed similar inhibition using generalised linear gamma model analysis. This could be attributed to the use of polyclonal antibodies rather than monoclonal ones, since only monoclonal antibodies prepared against the PPMV-1 variant virus can inhibit all PPMV-1 isolates in the HI test, but no other NDV strains can [68]. Monoclonal-specific epitopes on the F polypeptide induced greater neutralisation than those against the haemagglutinin-neuraminidase (HN) in vitro and in vivo tests [69]. 

Likewise, the two vaccination programs containing inactivated PPMV resulted in no pigeons shedding the virus. In contrast, the NDV LaSota vaccine did not prevent PPMV-1 virus shedding from the cloaca or orally, which was detectable from the 5th day post-challenge, comparable to other vaccinated groups. 

A notable problem of this disease is the virus shedding from the infected pigeons [49,51]. The main risk from the virus is its ability to transmit from infected birds to other bird populations (wild or domesticated), along with evolutionary changes, which potentially increases its virulence phenotype. So, protection against virus shedding is considered one of the important priorities for evaluating the success of vaccination programs. Nonetheless, the NDV LaSota vaccine provided insufficient protection against morbidity, mortality, and virus shedding, with low antibody levels pre- and post-challenge. Previous studies detected that the NDV-derived strain vaccines were inadequate for the protection of pigeons against PPMV-1 [35,70], as well as the Hitchner B1 vaccine [58]. Additionally, confirmed by field practices, it is shown that despite the use of the NDV LaSota vaccine in pigeons, the disease outbreaks continue [4,33], and this is the case in the pigeons from which the strain under study was obtained. This is attributed to the presence of biological, antigenic, and genetic differences between the NDV LaSota vaccine and the PPMV-1 strain [31,35,49,71], even if they are minor [33].

## 5. Conclusions

Applying live NDV chicken-adapted vaccines alone, such as LaSota, will not provide full protection against PPMV-1. This study provided a potential application of inactivated PPMV, which has the potential to provide full protection against PPMV-1. A more suitable and sufficient prophylactic program is the combination of both the live attenuated NDV vaccine, such as LaSota, and the inactivated PPMV-1 (G-VI). This diminishes the risk of both viruses from interspecies transmission. It ensures early protection against the challenges of field viruses. Along with this, in countries such as Egypt that have commercial PPMV vaccines, it is necessary to antigenically match them to currently circulating strains and address policymakers to implement a stricter control strategy. For more exploration, the efficacy of the vaccine programs applied in this study will be further investigated against other NDV genotypes in pigeons.

## Figures and Tables

**Table 1:** General and specific clinical signs, morbidities, and mortalities of differentially PPMV-1 virus-vaccinated groups 14 days post-challenge, with clarification of the healthy condition in the vaccinated, non-challenged groups.

Groups	Subgroups	General Signs *	Respiratory Signs	Greenish Diarrhoea	Nervous Signs	Morbidity (%)	Clinical Indices **	Mortality (%)
G1	A	0/8	0/8	0/8	0/8	0/8 (0)	0	0/8 (0)
	B	5/8	0/8	2/8	2/8	5/8 (62.5)	0.66	3/8 (37.5)
G2	A	0/8	0/8	0/8	0/8	0/8 (0)	0	0/8 (0)
	B	1/8	0/8	1/8	0/8	1/8 (12.5)	0.01	0/8 (0)
G3	A	0/8	0/8	0/8	0/8	0/8 (0)	0	0/8 (0)
	B	1/8	0/8	0/8	1/8	1/8 (12.5)	0.02	0/8 (0)
G4	A	0/8	0/8	0/8	0/8	0/8 (0)	0	0/8 (0)
	B	8/8	1/8	5/8	6/8	8/8 (100)	1.03	5/8 (62.5)

(*) General signs: ruffled feathers, lethargy, and anorexia. (**) Clinical indices: calculated as previously mentioned in the materials. Subgroups G1a, G2a, and G3a were vaccinated with live NDV LaSota, inactivated PPMV, and simultaneous NDV LaSota and PPMV vaccines, respectively, without challenge. Subgroups G1b, G2b, and G3b were vaccinated with live NDV LaSota, inactivated PPMV, and simultaneous NDV LaSota and PPMV vaccines, respectively, and challenged with PPMV-1 strain. However, G4a and G4b were kept non-vaccinated and non-challenged and challenged, respectively.

**Table 2:** Pathological lesions among the dead and euthanised morbid pigeons of differentially PPMV-1 virus-vaccinated groups post-challenge with the unvaccinated challenged group.

Subgroups	Total No.	No. of Dead and Euthanised Morbid Pigeons	Brain: Congested (C)/Haemorrhagic (H)	Spleen: Enlarged (E)/Atrophied (A)	Pancreas: Haemorrhages (H)/Necrosis (N)	Heart: Haemorrhages	Oesophagus to Proventriculus: Haemorrhages	Intestines: Enteritis with Greenish Content	Kidneys: Nephritis (Ni)/Nephrosis (No)	Thymus: Atrophy	Bursa of Fabricius: Atrophy
G1b	8	5	4 C	3	3 (1 H, 2 N)	0	1	3	3 (1 Ni, 2 No)	3	3
G2b	8	1	0	0	1N	0	0	1	0	0	0
G3b	8	1	1 C	0	1 N	0	0	0	1 No	0	0
G4b	8	8	8 C + H	5	8 (6 H, 2 N)	2	2	8	7 (2 Ni, 5 No)	5	4

Subgroups G1b, G2b, and G3b were vaccinated with live NDV LaSota, inactivated PPMV, and dual LaSota and PPMV vaccines, respectively, and challenged with PPMV-1. However, G4b was kept unvaccinated/challenged.

**Table 3:** Estimated marginal means ± SEM for the HI titre values over five weeks for five groups with two antigens.

Antigen	Group	Weeks Post-Vaccination				
		1	2	3	4	5
LaSota	G1b	1 ± 0.08 ^c^	1 ± 0.08 ^c^	1 ± 0.08 ^c^	1 ± 0.08 ^c^	4.67 ± 0.37 ^b^
	G2b	1 ± 0.08 ^c^	5 ± 0.39 ^b^	**6 ± 0.47 ^ab^**	6.33 ± 0.49 ^ab^	8.33 ± 0.66 ^a^
	G3b	1 ± 0.08 ^c^	1 ± 0.08 ^c^	5 ± 0.39 ^b^	5.67 ± 0.45 ^ab^	5 ± 0.39 ^b^
	G4a	1 ± 0.08 ^c^	1 ± 0.08 ^c^	1 ± 0.08 ^c^	1 ± 0.08 ^c^	1 ± 0.08 ^c^
	G4b	1 ± 0.08 ^c^	1 ± 0.08 ^c^	1 ± 0.08 ^c^	1 ± 0.08 ^c^	4.67 ± 0.37 ^b^
PPMV-1	G1b	1 ± 0.08 ^c^	1 ± 0.08 ^c^	1 ± 0.08 ^c^	1 ± 0.08 ^c^	6.33 ± 0.49 ^ab^
	G2b	1 ± 0.08 ^c^	4.33 ± 0.34 ^b^	**5.67 ± 0.45 ^ab^**	6.33 ± 0.49 ^ab^	8.33 ± 0.66 ^a^
	G3b	1 ± 0.08 ^c^	1 ± 0.08 ^c^	5 ± 0.39 ^b^	5.33 ± 0.42 ^ab^	5.67 ± 0.45 ^ab^
	G4a	1 ± 0.08 ^c^	1 ± 0.08 ^c^	1 ± 0.08 ^c^	1 ± 0.08 ^c^	1 ± 0.08 ^c^
	G4b	1 ± 0.08 ^c^	1 ± 0.08 ^c^	1 ± 0.08 ^c^	1 ± 0.08 ^c^	5.33 ± 0.42 ^b^

^abc^ Means with different superscript showed a statistical significance at *p* < 0.05. Bold values indicate the titre pre-challenge.

**Table 4:** Detection of viral shedding from collected swabs after the challenge of vaccinated and unvaccinated pigeons with the PPMV-1 isolate at 3, 5, and 7 days post challenge (dpc) and estimated by quantitative real-time PCR (RT-qPCR).

Group	Treatment	Tracheal Swab						Cloacal Swab					
		3 dpc		5 dpc		7 dpc		3 dpc		5 dpc		7 dpc	
		No. of Swabs *	Ct Value	No. of Swabs	Ct Value **	No. of Swabs	Ct Value **	No. of Swabs	Ct Value	No. of Swabs	Ct Value **	No. of Swabs	Ct Value **
G1b	LaSota vaccinated + challenged	0/3	-	1/3	37.6	1/3	30.9	0/3	-	2/3	32.6–34.9	1/3	28.4
G2b	PPMV vaccinated + challenged	0/3	-	0/3	-	0/3	-	0/3	-	0/3	-	0/3	-
G3b	LaSota and PPMV vaccinated + challenged	0/3	-	0/3	-	1/3	39.3 ***	0/3	-	0/3	-	1/3	37
G4b	Unvaccinated + challenged	0/3	-	1/3	37.3	3/3	35–36	0/3	-	3/3	32.8–33.9	3/3	33.9–35.4

(*) Three swab pools (two swabs/pool) for trachea and cloaca swabs, separately. (**) Average of the threshold cycle (Ct) values obtained by RT-qPCR according to the number of positive swabs and with indication to the amount of viral RNA. (***) Ct value > 39 was considered undetectable viral RNA.

## Data Availability

Participant data will be made available upon reasonable requests directed to the corresponding author. The investigator and collaborators will review and approve proposals based on scientific merit. After approval of a proposal, data can be shared through a secure online platform after signing a data access agreement.

## Supplementary Materials

The following supporting information can be downloaded at: https://www.mdpi.com/article/10.3390/v16101585/s1, Figure S1: Photomicrograph of control histopathology in different tissue organs of non-vaccinated non-challenged pigeons, H&E X400, Bar 20 µm; Figure S2: Photomicrograph of control immunohistochemistry (IHC) in different tissue organs of non-vaccinated and non-challenged (G4a) and challenged (G4b) pigeons with PPMV-1 strain, Bar 25 µm.
