# Supplementary material for: Assessment of PPMV-1 Genotype VI Virulence in Pigeons and Chickens and Protective Effectiveness of Paramyxovirus Vaccines in Pigeons
Source: Viruses. 2024 Oct 9;16(10):1585. doi: 10.3390/v16101585 (PMC11512342; doi:10.3390/v16101585)
Supplement: Supplementary file 1 [file viruses-16-01585-s001.zip › viruses-3197458-supplementary.pdf]

Supplementary materials

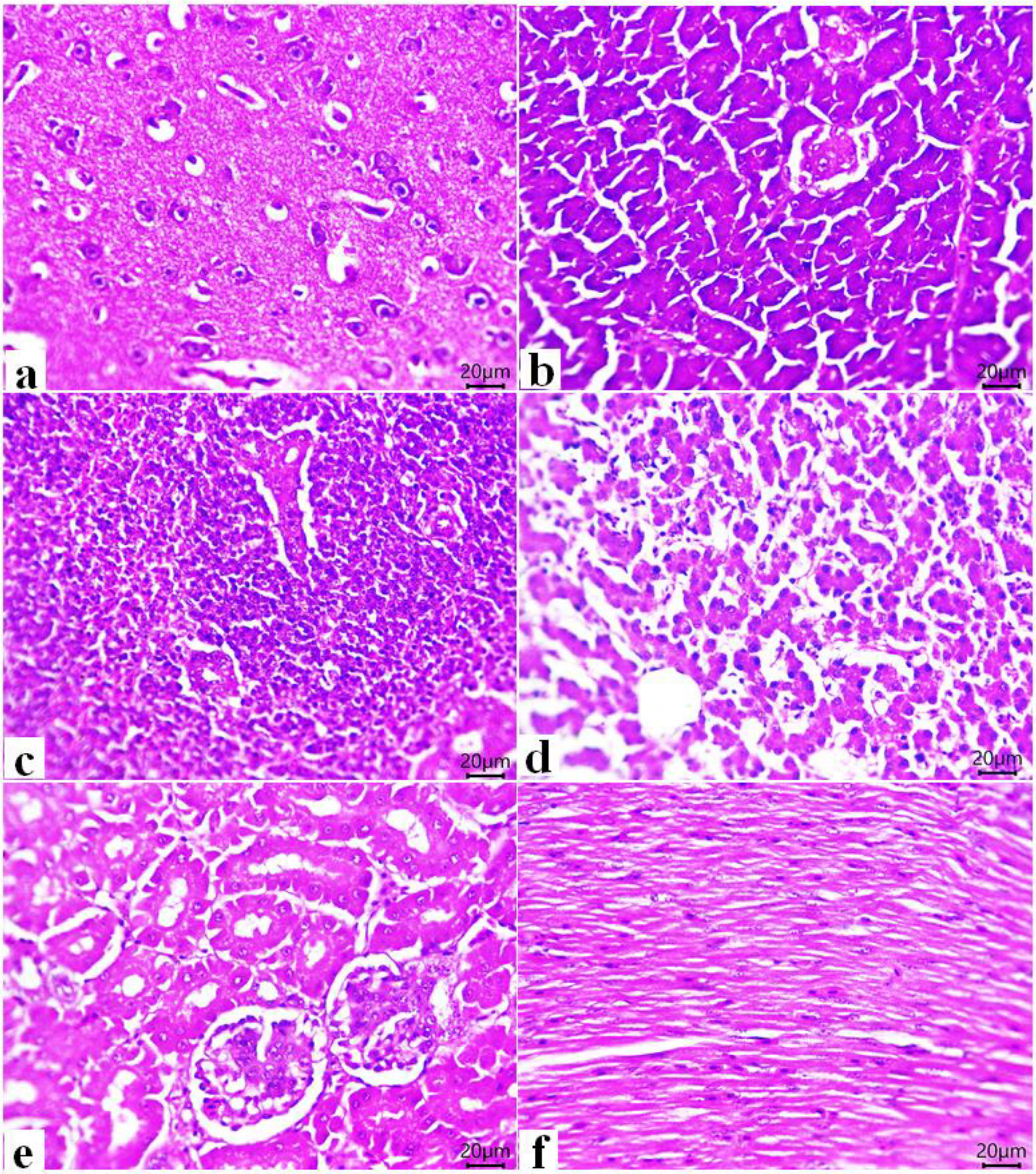

Supplementary Figure S1. Photomicrograph of control histopathology in different tissue organs of non-vaccinated non-challenged pigeons, H&E X400, Bar 20um. a) brain with normal histology of neurons, glia cells, neutrophil and vascular tissues, b) pancreas with normal morphology of pancreatic acinar epithelium and islets of Langerhans, c) spleen with normal cyto-architectures of white pulp lymphoid populations around central arterioles beside normal red pulp, d) liver with normal structures of hepatic acini, sinusoids, central vein, e) kidney with normal histological structures of glomerular tufts and renal tubules, and other stromal structures, f) heart with normal histology of cardiomyocytes with centrally located oval nuclei beside normal interstitial tissue.

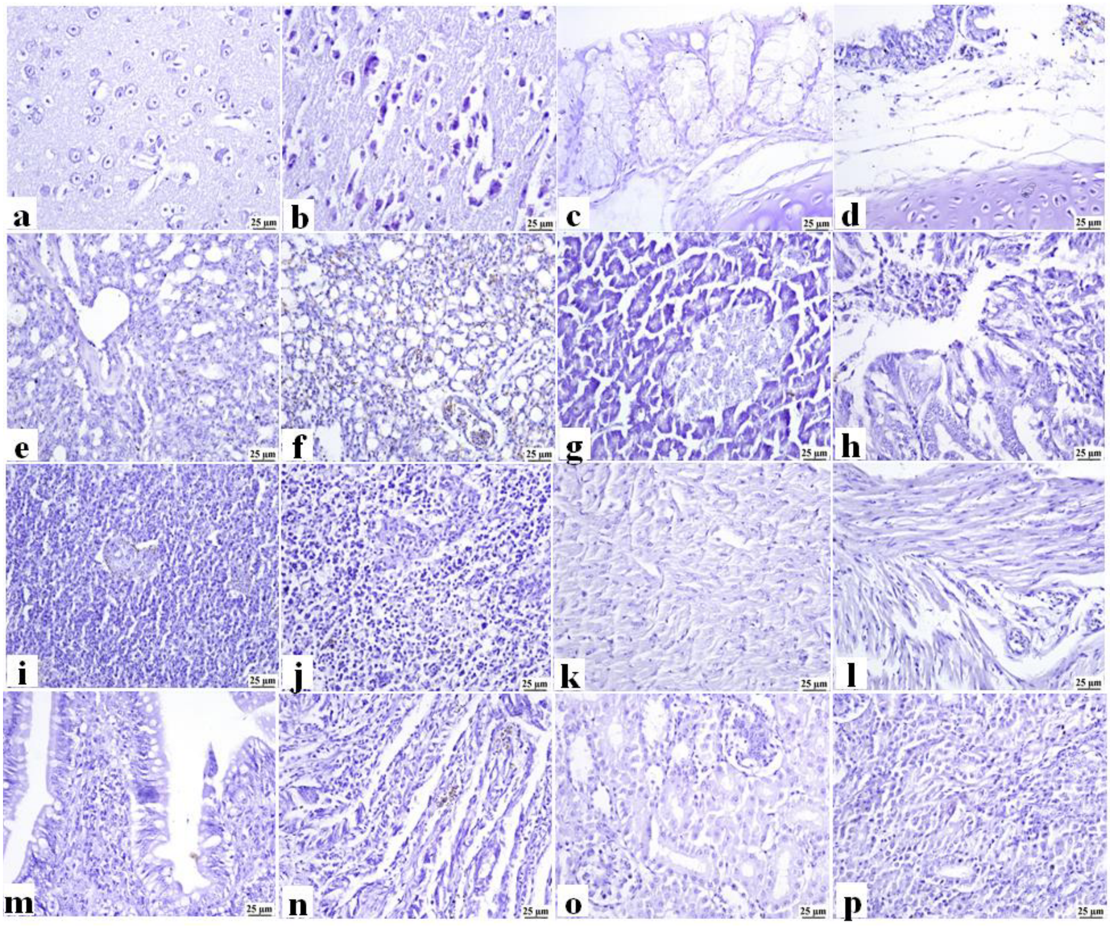

Supplementary Figure S2. Photomicrograph of control immunohistochemistry (IHC) in different tissue organs of non-vaccinated and non-challenged (G4a) and challenged (G4b) pigeons with PPMV-1 strain, Bar 25um. Negative expression of viral antigen in a) brain of non-vaccinated non-challenged pigeons, b) brain of non-vaccinated challenged pigeons, c) trachea of non-vaccinated non-challenged pigeons, d) trachea of non-vaccinated challenged pigeons, e) lung of non-vaccinated non-challenged pigeons, f) lung of non-vaccinated challenged pigeons, g) pancreas of non-vaccinated non-challenged pigeons, h) proventriculus of non-vaccinated challenged pigeons, i) spleen of non-vaccinated non-challenged pigeons, j) spleen of non-vaccinated challenged pigeons, k) heart of non-vaccinated non-challenged pigeons, l) heart of non-vaccinated challenged pigeons, m) intestine of non-vaccinated non-challenged pigeons, n) intestine of non-vaccinated challenged pigeons, o) kidney of non-vaccinated non-challenged pigeons, p) kidney of non-vaccinated challenged pigeons.

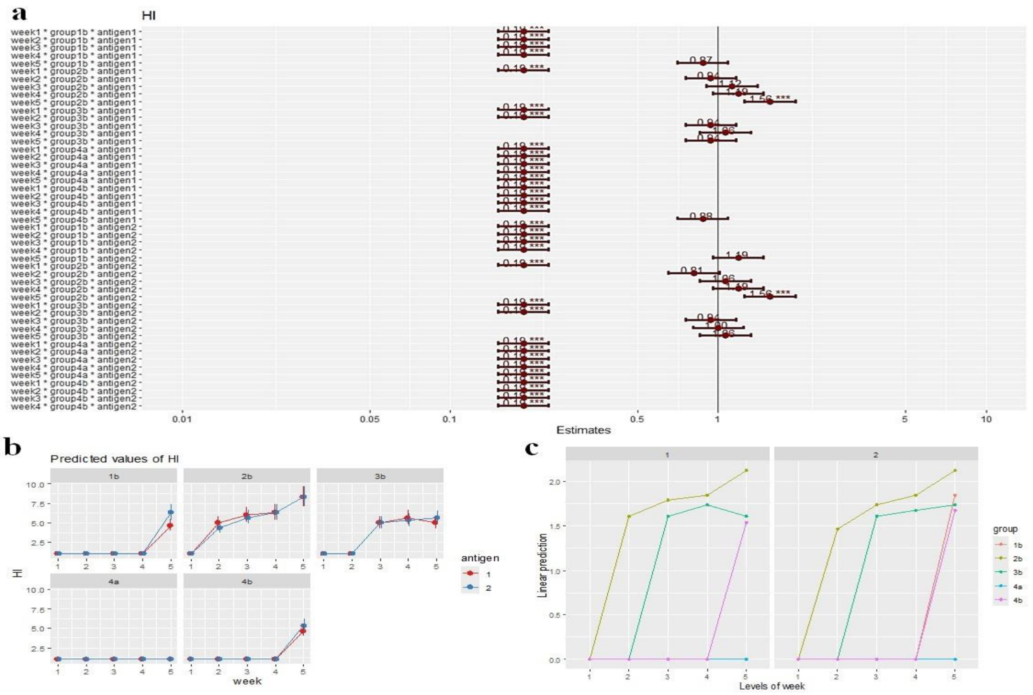

Supplementary Figure S3. (a) The generalised linear model (GLM) estimates with a 95% confidence interval.*** Represents the signifcance of the model coefficient estimates. (b) Predicted values of the GLM for HI titres to detect the difference between using LaSota as a heterologous antigen (antigen 1) and PPMV-1 as a homologous antigen (antigen 2) within the five groups. (c) Predicted values of the GLM for HI titres to detect the difference between the five groups within NDV LaSota (antigen 1) and PPMV-1 (antigen2) for five consecutive weeks.
